# *Pseudomonas putida* Responds to the Toxin GraT by Inducing Ribosome Biogenesis Factors and Repressing TCA Cycle Enzymes

**DOI:** 10.3390/toxins11020103

**Published:** 2019-02-09

**Authors:** Andres Ainelo, Rando Porosk, Kalle Kilk, Sirli Rosendahl, Jaanus Remme, Rita Hõrak

**Affiliations:** 1 Institute of Molecular and Cell Biology, University of Tartu, 51010 Tartu, Estonia; andres.ainelo@ut.ee (A.A.); sirliluup@gmail.com (S.R.); jaanus.remme@ut.ee (J.R.); 2 Institute of Biomedicine and Translational Medicine, University of Tartu, 50411 Tartu, Estonia; rando.porosk@ut.ee (R.P.); kmck@ut.ee (K.K.)

**Keywords:** toxin-antitoxin system, GraTA of HigBA family, *Pseudomonas putida*, whole-cell proteome, ribosome biogenesis, metabolome, TCA cycle

## Abstract

The potentially self-poisonous toxin-antitoxin modules are widespread in bacterial chromosomes, but despite extensive studies, their biological importance remains poorly understood. Here, we used whole-cell proteomics to study the cellular effects of the *Pseudomonas putida* toxin GraT that is known to inhibit growth and ribosome maturation in a cold-dependent manner when the *graA* antitoxin gene is deleted from the genome. Proteomic analysis of *P. putida* wild-type and Δ*graA* strains at 30 °C and 25 °C, where the growth is differently affected by GraT, revealed two major responses to GraT at both temperatures. First, ribosome biogenesis factors, including the RNA helicase DeaD and RNase III, are upregulated in Δ*graA*. This likely serves to alleviate the ribosome biogenesis defect of the Δ*graA* strain. Secondly, proteome data indicated that GraT induces downregulation of central carbon metabolism, as suggested by the decreased levels of TCA cycle enzymes isocitrate dehydrogenase Idh, α-ketoglutarate dehydrogenase subunit SucA, and succinate-CoA ligase subunit SucD. Metabolomic analysis revealed remarkable GraT-dependent accumulation of oxaloacetate at 25 °C and a reduced amount of malate, another TCA intermediate. The accumulation of oxaloacetate is likely due to decreased flux through the TCA cycle but also indicates inhibition of anabolic pathways in GraT-affected bacteria. Thus, proteomic and metabolomic analysis of the Δ*graA* strain revealed that GraT-mediated stress triggers several responses that reprogram the cell physiology to alleviate the GraT-caused damage.

## 1. Introduction

Prokaryotic toxin-antitoxin (TA) systems consist of two counterparts: A toxin that can inhibit central cellular processes, and an antitoxin that functions to neutralize the toxic effects. The toxins are small proteins, while the antitoxins can function as either proteins or RNAs. The nature and specific mode of action of the antitoxin is the basis for TA systems’ division into seven different types [1]. Of these, the most thoroughly studied is type II, where the antitoxin is a small protein and inhibits the toxin by directly binding it in an inactive complex. TA toxin activation is mediated by the differential stability of the two proteins; the antitoxins are generally less stable than the toxins. This results in their preferential degradation upon protease activation or loss of TA genes, for example in the case of TA-carrying plasmid loss [2].

These systems are prevalent not only on plasmids but also in bacterial genomes [3,4], where the question of their biological meaning and relevance has induced an intense debate. Due to their self-stabilizing nature, it has been proposed that TA modules are just selfish remnants of mobile genetic elements [5]. Still, specific TA systems have been associated with several physiological roles, which suggests that they may be integrated into the host’s regulatory network. For example, the *Escherichia coli* MqsR/MqsA TA system has been implicated in the oxidative stress response, regulating biofilm formation [6] and enhancing bile acid stress tolerance [7]. MqsR activation, in turn, results in the degradation of the GhoT/GhoS TA system antitoxin mRNA, demonstrating the TA systems’ potential for cross-activation [8]. Several TA systems have been linked to the formation of persister cells, a dormant subpopulation that is not killed by antibiotics. While the model that depicted TA systems as the key players in *E. coli* persistence through polyphosphate activation of Lon protease turned out to be an experimental artifact [9,10], there are still individual works that demonstrate TA’s relevance in the persistence under certain conditions [11,12,13,14,15].

Most likely due to their frequent association with mobile genetic elements that can transfer between diverse bacterial species, the studied TA toxins all target essential and conserved cellular structures and processes. Several toxins disturb DNA metabolism [16,17,18] and the cell envelope [19,20,21]. However, most toxins attack the translational apparatus, employing an impressive variety of mechanisms [22,23]. Kinase toxins have been found to phosphorylate the glutamyl-tRNA synthetase and EF-Tu [24,25,26,27] while the GNAT-fold toxins acetylate tRNAs [28,29]. A large number of toxins function as RNases, collectively able to cleave every major RNA species: tRNAs [30,31], rRNAs both as pre-rRNA [32,33] and in the context of the ribosome [34,35], and mRNAs both in their free state [36,37,38,39,40] or co-translationally in a ribosome-dependent fashion [41,42,43,44,45].

The most thoroughly studied TA system in the metabolically versatile soil bacterium *Pseudomonas putida* is the type II GraT/GraA module [46]. It is homologous to the HigB/HigA systems, where the toxin is a ribosome-dependent mRNase [47]. However, GraT stands out among them due to its conditional toxicity: At the *P. putida* preferred growth temperature of 30 °C, the toxic effects are so mild that the antitoxin gene *graA* can be deleted from the genome with only a marginal growth defect. Lowering the temperature progressively enhances the toxicity so that at 20 °C, the antitoxin deletion strain Δ*graA* has a severely reduced growth rate in liquid medium and is unable to form colonies on solid medium [46]. Like other HigB family toxins, GraT cleaves mRNAs in a codon-specific fashion with relatively low sequence specificity. The only consistent feature of the cut sites is an adenine in the 2nd position of the codon [48]. Considering that GraT functions as ribosome-dependent mRNase, it is intriguing that one of its physiological effects is the inhibition of ribosome biogenesis, as evidenced by the accumulation of nearly complete ribosomal subunits in the cells [49]. Conversely to GraT, the expression of homologous ribosome-associated mRNases HigB and RelE has been demonstrated to reduce the amount of free ribosomal subunits [47] instead of their accumulation. Structural analysis revealed another feature of GraT that distinguishes it from other HigB family toxins. While the HigB toxins are fully folded proteins [50,51,52], the N-terminus of GraT is not resolved in the crystal structures. This disordered region plays a dual regulatory role in controlling both *graTA* operon expression and GraT toxicity [48]. Intriguingly, the central chaperone of protein folding, DnaK, is implicated in GraT toxicity [49]. While not verified yet, DnaK most probably assists with the folding of GraT structure.

Thus, GraT seems to have several unusual features (temperature-dependence effects, structural disorder) and outcomes to cell physiology (ribosome biogenesis defect) that discriminate it from other HigB toxins. We are especially interested in the physiological consequences of GraT-mediated mRNA degradation that culminates with the ribosome biogenesis defect. The antitoxin deletion strain Δ*graA* is a valuable tool as GraT toxicity can be modulated by the growth temperature [46,49]. This is in contrast with the conventional overexpression technique utilized in studying TA toxin effects. While useful for determining toxins’ molecular targets, toxin overexpression is an artificial system that does not necessarily mimic genomic TA activation conditions. Antitoxin deletion from the TA locus is a closer approximation of native TA system activation, and thus, it is a good model system to study the potential physiological effects of a toxin.

The current study aimed to identify the whole-cell-level consequences of GraT toxicity by comparing the proteomic profiles of *P. putida* wild-type and Δ*graA* strains. Proteome analysis revealed upregulation of several ribosome biogenesis factors in Δ*graA,* suggesting that a specific response is induced to alleviate the GraT-caused ribosome biogenesis defect. Proteome data also indicated that GraT inhibits central carbon metabolism, as several TCA cycle enzymes were downregulated in the Δ*graA* strain. The metabolite analysis revealed that the TCA intermediate oxaloacetate accumulates in Δ*graA*, further confirming the metabolic reprogramming in response to GraT activity.

## 2. Results and Discussion

### 2.1. Whole-Cell Proteomic Response to GraT

The GraT-caused cold-sensitive growth [46] and ribosome maturation defects [49] are not common phenotypes among TA toxins, and only a few other TA systems are reported to be affected by temperature [53,54,55,56]. To gain more insights into the cellular effects of GraT, we compared the whole cell proteome of the Δ*graA* strain with the *P. putida* wild-type. We expected that proteome level analysis will uncover the ribosome biogenesis factor(s) downregulated due to GraT activity and additionally reveal the cellular response that counters GraT toxicity.

**Figure 1:**
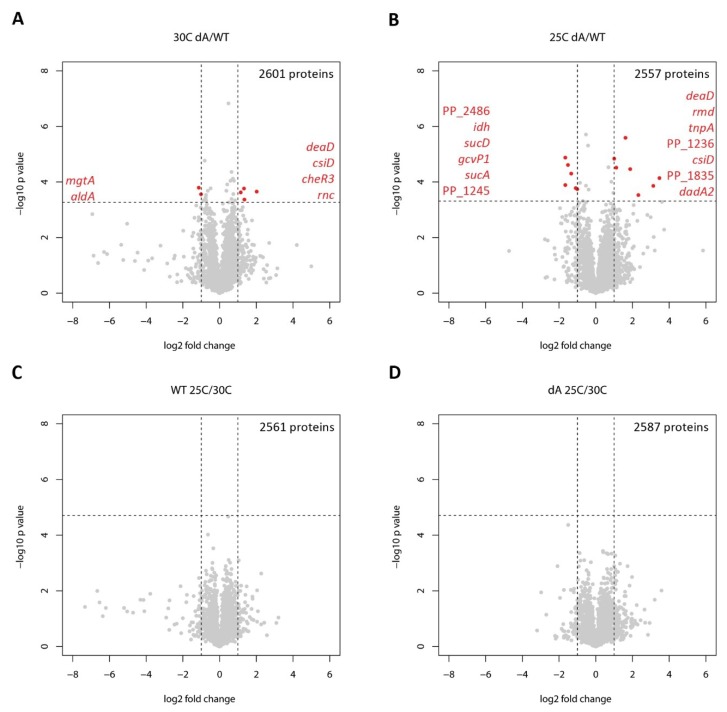
Overview of *P. putida* wild-type and Δ*graA* proteome comparisons at 30 °C and 25 °C. Volcano plots represent comparisons of Δ*graA* to the wild-type at 30 °C (**A**) and 25 °C (**B**), and the temperature-dependent proteomic response of the wild-type (**C**) and the Δ*graA* strain (**D**). The number of proteins in each comparison is indicated in the respective plots. The horizontal dashed lines indicate the statistical significance thresholds after Benjamini-Hochberg multiple testing correction (FDR = 0.05). Vertical dashed lines indicate the twofold difference between the compared proteomes. Proteins with statistically significant and more than twofold changes are represented by red dots and listed in order of decreasing statistical significance (increasing *p*-values).

Bacteria for proteome analysis were grown to the mid-exponential phase both at 30 °C, where the toxin causes only a slight growth defect (generation time of Δ*graA* strain compared to wild-type is 37% longer), and 25 °C, where the generation time of Δ*graA* strain was increased by 72% (Figure S1). Differences between proteomes of wild-type *P. putida* and the Δ*graA* mutant were detected already at 30 °C. Of the 2601 proteins compared, 25 were significantly and differently expressed in the Δ*graA* strain (Table S1), and 6 of those varied more than 2-fold relative to the wild-type (Figure 1A and Table 1). Thus, although the growth defect caused by GraT is rather small at 30 °C, it is accompanied by a measurable proteome alteration. Considering this result and the significantly decreased growth rate of the Δ*graA* strain at 25 °C, it was, therefore, surprising that the overall proteome response of the Δ*graA* strain had only slightly amplified at 25 °C; analysis of 2557 proteins revealed 21 proteins that were significantly differentially expressed in Δ*graA* (Table S2), and 13 of those differed more than 2-fold (Figure 1B and Table 1).

When we looked at temperature-dependent changes within each strain, none of the protein variations remained statistically significant after multiple testing correction was applied (Figure 1C,D). This indicates that adaptation to the temperature shift from 30 °C to 25 °C is not accompanied by major changes in the proteome either in the wild-type or Δ*graA* strain but is rather characterized by smaller changes that do not cross the individual significance threshold. Also, even though the GraT-induced changes occur in the Δ*graA* strain at both 30 °C and 25 °C (Figure 1A,B and Table 1), these changes at each temperature do not differ principally, and proteome alterations starting at 30 °C will just gradually increase at 25 °C (see below).

Applying multiple testing correction assumes that all the tests are totally independent of each other. However, many prokaryotic genes are co-transcribed as a single mRNA and are thus coupled in their expression. To somewhat account for this biological linkage, we combined the comparisons between two strains with the list of predicted *P. putida* operons from the DOOR database [57]. Searching for operons where the mean change was more than twofold yielded 7 down- and 5 upregulated operons in Δ*graA* at 30 °C and 11 down- and 12 upregulated operons at 25 °C (Table S3).

### 2.2. Proteome Changes Indicating the Cellular Response to the Ribosome Biogenesis Defect of the ΔgraA Strain

**Figure 2:**
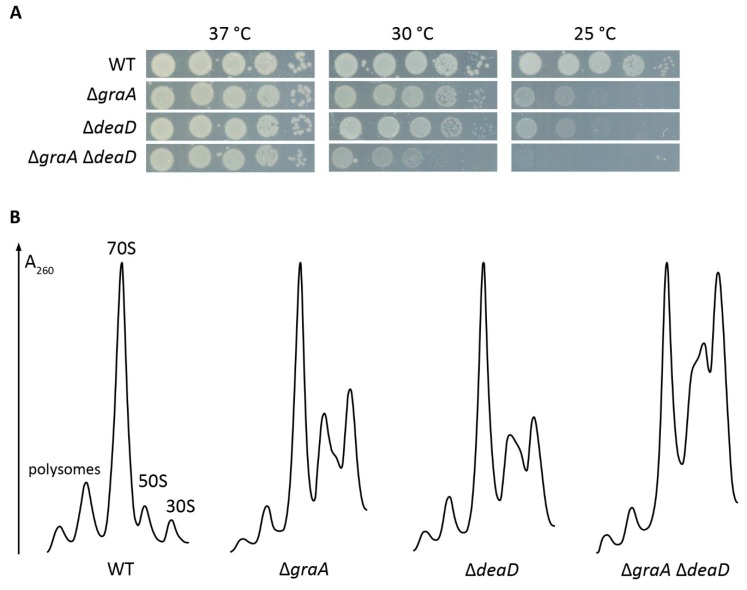
The effects of *deaD* deletion from the *P. putida* wild-type and Δ*graA* strains. (**A**) Temperature-dependent growth assay. Tenfold dilution series were spotted on LB plates and incubated for 24 h at the indicated temperature. (**B**) Sucrose gradient centrifugation profiles of ribosomal particles from cultures growing exponentially (OD_580_ ≈ 1.0) at 25 °C. Profiles were normalized to 70S peak heights.

Considering that GraT induces a ribosome biogenesis defect, we expected the proteome analysis to reveal the decreased abundance of some ribosome biogenesis factor(s) in the Δ*graA* strain. However, no known ribosome biogenesis proteins were detected to be statistically significantly downregulated in Δ*graA*. On the contrary, significant upregulation of the RNA helicase DeaD/CsdA (Cold-shock DEAD-box protein A), a known ribosome assembly factor in *E. coli* [58,59,60], was observed in Δ*graA* at both 25 °C and 30 °C (Table 1). Increased expression of DeaD could be considered as a stress response to alleviate the GraT-induced ribosome biogenesis defect. Thus, to investigate the importance of DeaD in GraT-caused phenotypes, we constructed the Δ*deaD* and the Δ*graA*Δ*deaD* strains and tested their growth at different temperatures. Data show that deletion of *deaD* from the wild-type *P. putida* causes a similar growth defect to the Δ*graA* strain on solid medium at 25 °C (Figure 2A). This is consistent with *E. coli deaD*, which also causes a cold-sensitive phenotype when deleted [59]. The *graA* and *deaD* double deletion shows a cumulative effect as the Δ*graA*Δ*deaD* strain grows even more poorly than either single deletion strain. At 37 °C, however, the mutants grow as well as the wild-type (Figure 2A).

We also compared the ribosomal particle profiles of the DeaD deletion strains with the wild-type and Δ*graA* strains grown at 25 °C. Both Δ*graA* and Δ*deaD* show subunit accumulation that is characteristic of a ribosome biogenesis defect (Figure 2B). However, the profiles differ between the mutant strains, as Δ*deaD* shows slightly fewer 50S and 30S subunits and more intermediate particles sedimenting at around 40S than the Δ*graA* strain (Figure 2B). These results indicate that the role of DeaD is similar in *P. putida* and *E. coli*, as the *E. coli* DeaD-deficient strain that also displays intermediate particles of approximately 40S [58]. Also considering that the Δ*graA*Δ*deaD* strain displays a cumulative growth defect that is reflected in the severely disturbed ribosomal particle profile (Figure 2A,B), the overproduction of DeaD is most likely a mechanism for Δ*graA* cells to cope with the GraT-inflicted ribosome biogenesis inhibition.

Interestingly, other ribosome biogenesis-associated proteins besides DeaD tend to be upregulated as well in Δ*graA*. The rRNA-processing RNase III [61] was significantly 2.6-fold upregulated in Δ*graA* already at 30 °C (Table 1). The RNase III gene is part of the two-gene *rnc-era* (PP_1433-PP_1434) operon which also codes for the GTPase Era, another known ribosome biogenesis factor [62]. The operon analysis revealed upregulation of the whole *rnc-era* operon at 30 °C with the levels of Era increasing 1.8-fold (Table S3). Additionally, the abundances of RNase III and Era were increased about 3.3 and 1.7-fold, respectively, at 25 °C (Table S3). Although the single changes were statistically insignificant after multiple testing correction (p=0.0025 and p=0.011, respectively), the same trend of change of the two proteins suggests a regulated response of the whole operon.

At 25 °C, we also identified a four-gene operon, PP_4787-PP_4790, where the three first genes were up to 3-fold upregulated (Table S3). The second gene in the operon codes for the endoribonuclease YbeY, which is involved in 16S rRNA maturation and 70S ribosome quality control in *E. coli* [63], though its exact role is unclear [64]. The first gene in the operon, *ybeZ*, encodes a protein with an unknown function. However, the findings that *E. coli* YbeZ interacts with YbeY [65] and ribosomal proteins S7 and L6 [66] link YbeZ with ribosome biogenesis as well.

**Figure 3:**
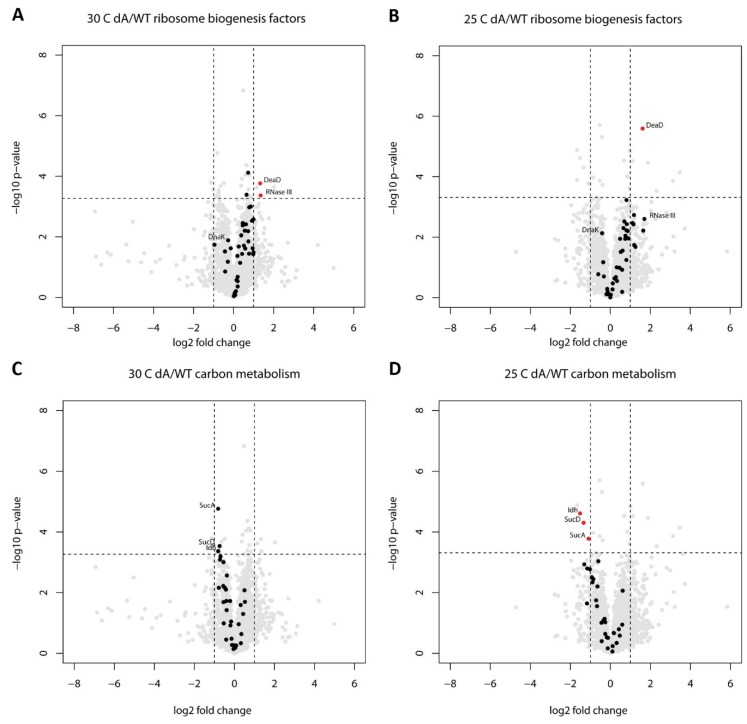
Volcano plots representing the comparisons of the *P. putida* Δ*graA* strain to the wild-type at 30 °C and 25 °C with ribosome biogenesis factors (**A**,**B**) or carbon metabolism enzymes (**C**,**D**) highlighted as black dots. The highlighted proteins are listed in Table S3. Horizontal dashed lines indicate the statistical significance thresholds after Benjamini-Hochberg multiple testing correction (FDR = 0.05). Vertical dashed lines indicate a twofold difference between the compared proteomes.

We also looked for other known ribosome biogenesis factors in our between-strains comparisons to potentially find signs of concerted regulation. Among the 39 ribosome biogenesis factors (the list was compiled from [67,68,69]), the major trend is indeed towards upregulation (Figure 3A,B). Only the chaperones DnaK/J and GroEL/ES stand out with a trend for downregulation (Table S4), although not very strongly. Similar trends in ribosome biogenesis factor levels were observed for both 30 °C and 25 °C (Figure 3A,B), which increases confidence in that these individually mostly nonsignificant changes are not just experimental noise.

Taken together, the significant upregulation of the RNA helicase DeaD and RNase III, as well as the trend towards a higher abundance of several other ribosome biogenesis factors, suggest that the GraT-caused damage induces a specific response intended to alleviate the ribosome biogenesis defect of the Δ*graA* strain.

### 2.3. Proteome Changes Reveal Altered Carbon Metabolism of the ΔgraA Strain

**Figure 4:**
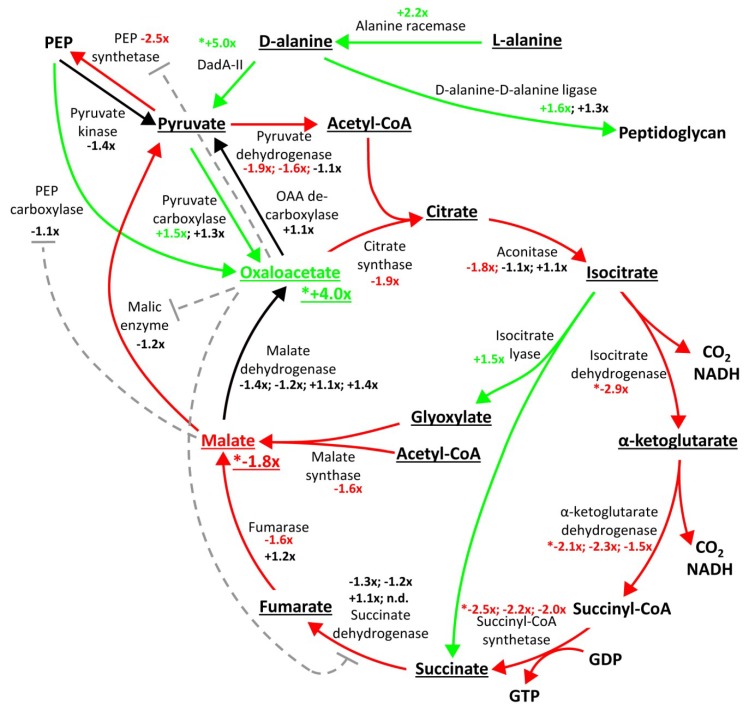
Schematic overview of the TCA cycle and related enzyme level and metabolite level changes in *P. putida* Δ*graA* compared to the wild-type at 25 °C. Underlined compound levels were measured by mass spectrometry. Asterisks denote statistically significant changes. Dashed lines indicate allosteric repression by oxaloacetate or malate. Red lines indicate reactions where an enzyme subunit is at least 1.5-fold downregulated or that is subject to repression by changes in oxaloacetate (OAA) levels. Green arrows indicate reactions where an enzyme subunit is at least 1.5-fold upregulated or that is subject to lowered repression by malate.

Besides proteomic alterations associated with ribosome biogenesis, we also detected several metabolism-related changes induced by GraT. Notably, three of the six significantly downregulated proteins in the Δ*graA* strain at 25 °C are the TCA cycle enzymes isocitrate dehydrogenase Idh, α-ketoglutarate dehydrogenase subunit SucA, and succinate-CoA ligase SucD subunit (Table 1). The levels of these enzymes responsible for converting isocitrate to succinate (Figure 4) were found to have dropped more than 2-fold (Table 1). Downregulation of these proteins suggests that particularly decarboxylation and the substrate-level phosphorylation steps of the TCA cycle are decreased in GraT-affected bacteria. However, when looking at other TCA cycle enzymes, they also tend to be downregulated in the Δ*graA* strain, although the changes are statistically nonsignificant (Figure 3D, Figure 4). The same overall trend of TCA enzymes is observable at 30 °C (Figure 3C, Table S4).

The other three significantly less abundant proteins in the Δ*graA* strain grown at 25 °C are the glycine dehydrogenase GcvP, a PP_2486-encoded putative NADH-dependent oxidoreductase, and a hypothetical protein encoded by PP_1245 (Table 1). GcvP is part of a multienzyme glycine-cleavage system (GCS or GCV) that catalyzes glycine decarboxylation to form 5,10-methylenetetrahydrofolate, an important cofactor in one-carbon metabolism [70]. Notably, this is the third significantly downregulated protein that participates in reactions resulting in CO_2_ release. In line with GcvP downregulation, the combined proteome and operon analysis suggests that the other GCS proteins, GcvH (Table S3) and GcvT (Table S2), are also about 2-fold downregulated in the Δ*graA* strain at 25 °C, although the changes are not significant (p = 0.020 and p = 0.0007; respectively). Interestingly, a putative GCV system transcriptional repressor encoded by PP_1236 is among the significantly upregulated proteins in Δ*graA* at 25 °C (3.7-fold, Table 1), which may explain the GCV system downregulations. Although this protein has not been studied in *Pseudomonas*, the corresponding *E. coli* GcvR binds to the transcriptional activator GcvA and reverses its function, causing transcriptional repression of the *gcv* operon [71]. Glycine functions as a derepressor by binding GcvR and disrupting its binding to GcvA [72]. Intriguingly, inactivation of *gcvP* or *gcvT* has been recently shown to suppress the toxicity of the *E. coli* YafQ [56]. Thus, the activity of the GCV system seems to be either unnecessary or even detrimental to *P. putida* and *E. coli* during activation of ribosome-dependent mRNAse toxins such as GraT and YafQ. In our transposon mutagenesis experiment to find GraT suppressor mutants [49], we detected no insertions into *gcv* genes. It still remains possible that the GCV downregulation functions to counter GraT toxicity, but at a level that remained below the transposon assay detection threshold.

Contrarily to the TCA enzymes downregulated in the Δ*graA* strain, some other metabolism proteins were significantly upregulated in response to GraT. For instance, the levels of the D-amino acid dehydrogenase DadA and the GDP-mannose 4,6 reductase Rmd had increased 5- and 2-fold, respectively, in Δ*graA* grown at 25 °C (Table 1). In *P. aeruginosa*, Rmd is required for synthesis of GDP-D-rhamnose [73,74] and DadA for utilization of alanine [75]. While DadA mainly deaminates D-alanine to pyruvate (Figure 4), it also participates in the catabolism of other D-amino acids such as valine, serine, threonine, phenylalanine, and histidine [76]. D-alanine is an important precursor in peptidoglycan synthesis and is made from L-alanine by alanine racemases [77]. Interestingly, the amount of Alr alanine racemase (also known as DadX), encoded by the gene downstream of *dadA*, PP_5269, was also 2-fold increased in the Δ*graA* strain at 25 °C (Table S2). Even though this change was statistically insignificant (p = 0.038), this suggests that the D-alanine metabolism seems to be elevated in the Δ*graA* strain. The reason for this regulation is not clear, but one may hypothesize that the induction of D-alanine metabolism would be helpful to counteract the GraT-caused membrane permeability defect [46].

CsiD (also Gab or YgaT in *E. coli*), the carbon starvation-induced protein in *E. coli* [78], was the most strongly upregulated (11-fold at 25 °C, 4-fold at 30 °C) significantly changed protein in Δ*graA* (Table 1). Additionally, the protein encoded by its following *lhgO* gene was 12-fold upregulated at 25 °C (3-fold at 30 °C), although the p-value (0.00052) fell just below the significance level (Table S2). This indicates that *csiD* and *lhgO* are likely co-expressed although they are not predicted to compose an operon in the DOOR database [57]. The proteins coded by subsequent genes in the *csiD-lhgO* locus were either not detected or their levels had not changed in the Δ*graA* strain.

In *E. coli*, the *csiD* operon has been studied as an example of a strictly RpoS-controlled transcriptional unit [78]. Yet, relatively little is known about the function of the protein itself. Based on structural analysis, it resembles α-ketoglutarate oxygenase, but it does not bind α-KG in its active site [79]. LhgO (*E. coli* YgaF) functions as a L-2-hydroxyglutarate oxidase that is able to regenerate mistakenly reduced α-ketoglutarate [80]. This led us to hypothesize that the strong upregulation of CsiD and LhgO could be a counter-effect that relieves the GraT-caused metabolic stress indicated by downregulation of the TCA cycle. Thus, we deleted both genes individually from both the wild-type and the Δ*graA* strain. Analysis of mutants’ growth at different temperatures showed, however, that the lack of either CsiD or LhgO did not affect the cold-sensitive growth defect of the Δ*graA* strain (data not shown). Neither did CsiD and LhgO deficiency affect the growth of Δ*graA* under stress conditions such as exposure to antibiotics (ciprofloxacin, benzylpenicillin, kanamycin, tetracycline, streptomycin, rifampicin) or chemicals such as nitroquinoline, paraquat, and NaCl (data not shown). Thus, unlike the increased expression of ribosome biogenesis factors in the Δ*graA* strain, the upregulation of CsiD and LhgO cannot be considered as a response dedicated to alleviating the GraT-inflicted defects. Given that in *E. coli*, the *csiD* and *lhgO* genes are induced upon carbon starvation [81], it is possible that GraT causes a similar carbon starvation-like situation for *csiD* and *lhgO* upregulation. However, as levels of RpoS were not significantly changed in the Δ*graA* strain, the increased expression of the *csiD* operon cannot be explained by RpoS-dependent activation, as described in *E. coli* [78].

### 2.4. Metabolomic Analysis Indicates Accumulation of Oxaloacetate in the ΔgraA Strain

Given the slower growth rate of GraT-affected bacteria, the decrease in central carbon metabolism is not surprising. Still, the proteome data does not allow us to conclude whether this is just a consequence of the lowered translation of the toxin-stressed bacteria or if GraT targets some certain step(s) of the TCA cycle. To gain more insights into the metabolic state of the Δ*graA* cells, the metabolic alterations in the wild-type and Δ*graA* were studied at 25 °C and 30 °C. Namely, TCA cycle intermediates, hydroxyl acids, and amino acids were analyzed by LC-MS.

Mainly isocitrate dehydrogenase, but also α-ketoglutarate dehydrogenase and citrate synthase, are the rate-limiting enzymes of the TCA cycle. Considering that the isocitrate and α-ketoglutarate dehydrogenases, as well as succinyl-CoA synthetase, were strongly downregulated in the Δ*graA* strain at 25 °C (Table 1), it was somewhat surprising that the levels of both citrate and isocitrate remained on par with respective values in the wild-type (Figure 4, Table S5). However, as the proteome data indicates that the isocitrate lyase AceA is 1.5-fold upregulated (*p* = 0.008) in Δ*graA* (Table S2, Figure 4), the shortcut from isocitrate to succinate over glyoxylate is possibly elevated, removing the bottleneck from downregulated dehydrogenases. Metabolomic analysis revealed only two statistically significant changes of TCA cycle intermediates: Oxaloacetate (OAA) levels had increased 4-fold (*p* = 2.5 × 10^−7^) and malate levels had decreased 1.8-fold (*p* = 0.0008) in the Δ*graA* strain at 25 °C (Table S5 and Figure 4). At the higher temperature, a similar increase in OAA levels was observed, although it was less pronounced and not statistically significant (Table S5). This suggests that already at 30 °C when bacterial growth is not yet inhibited by GraT, the toxin actually triggers essential changes in bacterial central metabolism. The reason for the OAA accumulation could be the decreased flux through the TCA cycle due to the downregulation of most TCA enzymes (Figure 4). Still, the remarkable exceptions are oxaloacetate-producing enzymes: Malate dehydrogenase, pyruvate carboxylase, and PEP carboxylase show no significant changes in protein levels (Figure 4). Anaplerotic production of OAA from PEP could even be higher in the Δ*graA* strain, as there is less malate, which is a known allosteric inhibitor of PEP carboxylase activity [82]. OAA formation from pyruvate is probably also increased, particularly when considering that pyruvate production from D-alanine may be increased due to the 5-fold higher abundance of the DadA (Table 1). Oxaloacetate also allosterically affects the enzymes of the TCA cycle and related pathways in *E. coli*. OAA is known to inhibit the malic enzyme MaeB [83], PEP synthetase [84] and the succinate dehydrogenase complex [85]. Notably, only one of these four allosterically regulated enzymes, PEP synthetase PpsA, is also 2.5-fold downregulated (*p* = 0.0011) in the Δ*graA* proteome (Table S2 and Figure 4). The other three enzymes affected by OAA or malate are among the TCA-related proteins that showed very little differential regulation in the Δ*graA* strain.

We detected no meaningful amino acid level changes in Δ*graA* at either 30 °C or 25 °C. While some comparisons did present statistically significant differences, the changes remained below 1.5-fold and thus we did not pursue them further (Table S5).

Combining the proteomics with metabolite measurements reveals slowdown of the whole tricarboxylic acid cycle in the Δ*graA* strain, with most enzymes being either less abundant or allosterically inhibited by the increased concentration of OAA (Figure 4). The main exceptions are oxaloacetate-producing enzymes, which most probably contribute to OAA accumulation. However, OAA is not only the intermediate of the TCA cycle, but also an important branch point towards anabolic pathways with about half of OAA flowing out of the TCA cycle [86]. Accumulation of OAA in Δ*graA* strain, therefore, also suggests that the anabolic flux from the oxaloacetate node should be decreased in GraT-affected bacteria. Thus, accumulation of OAA most probably signals that both catabolic and anabolic pathways are inhibited in GraT-affected bacteria.

## 3. Conclusions

This study described the proteomic and metabolomic alterations in *P. putida* caused by the chromosomal *graT* toxin gene when the GraA antitoxin is missing. One copy of the *graT* only slightly impedes the growth rate of the Δ*graA* strain at 30 °C and allows growth at 25 °C with the growth rate about two-fold lower than the wild-type [46]. Thus, differently from many other studies where the consequences of a TA toxin are studied by artificial overexpression of the toxin that often arrests bacterial growth, we analyzed the toxin effects in growing bacteria. We consider this difference to be most important because this allowed us not only to study the damage the toxin is causing but also to detect the physiological response of bacteria to the toxin-caused stress. We consider this more relevant to natural conditions where one chromosomal copy of a toxin can occasionally escape from the antitoxin-mediated control.

Proteome data obtained in the current study corroborates the previous finding that GraT inhibits ribosome biogenesis [49]. This was evidenced by the upregulation of several ribosome biogenesis factors, including RNA helicase DeaD and RNase III, which most probably alleviate the GraT-caused ribosome biogenesis defect. Thus, GraT activity seems to induce a specific response meant to antagonize the damage that the toxin is producing. Interestingly, while the response against the toxin-caused ribosome biogenesis defect was recognized, no clear reason why the ribosome biogenesis is deficient was deciphered from the Δ*graA* proteome. However, this might not be surprising, because GraT is a ribosome-dependent endoribonuclease that has low sequence specificity [48] and most probably cleaves numerous translated mRNAs. Therefore, we propose that the defect in ribosome biogenesis of the Δ*graA* strain is a cumulative effect resulting from the degradation of multiple GraT targets. Intriguingly, the endoribonuclease toxin MazF, that differently from GraT cleaves RNAs independent of the ribosome, was recently shown to inhibit rRNA maturation and ribosome biogenesis as well [33]. The authors concluded that this probably results from MazF-mediated cleavage of rRNA precursors and ribosomal protein transcripts. Still, the ribosomal RNAs are not likely substrates for GraT because this toxin cleaves mRNAs in a ribosome-dependent manner [48] and, furthermore, ribosomal RNAs were shown to be intact in the Δ*graA* strain [49]. GraT seems also not to have major effects on the abundance or stoichiometry of ribosomal proteins because proteome data disclosed no difference in the ribosomal protein abundance in *P. putida* wild-type and Δ*graA* strains (Figure S2).

The proteome data also revealed that in response to the toxin GraT, central carbon metabolism is downregulated as evidenced by the decreased levels of several TCA cycle enzymes. We propose that the metabolism downregulation has two reasons. First, GraT can directly impair cell metabolism by targeting the mRNAs of metabolic enzymes. However, it is even more plausible that the observed metabolic alterations represent an adaptive readjustment of cell physiology in response to inhibition of ribosome biogenesis and translation. Interestingly, the significantly decreased TCA enzyme levels were not accompanied by changes in the corresponding intermediate compounds, indicating the importance of maintaining their constant levels (Figure 4). The remarkable exceptions were, however, oxaloacetate and malate, as OAA levels were drastically increased and malate levels significantly decreased in the Δ*graA* strain. The reasons for the OAA accumulation could be both the decreased flux through the TCA cycle and likely also the reduced usage of OAA in biosynthetic processes. The latter would result from impairment of anabolic pathways due to GraT-mediated mRNA cleavages.

Taken together, the proteomic and metabolomic analysis of the Δ*graA* strain identified several cellular responses triggered by the toxin GraT. Some alterations, such as upregulation of ribosome biogenesis factors, evidently alleviate the GraT-caused damage. However, the metabolism reprogramming might also be of adaptive value by adjusting the cell physiology to balance the toxin-mediated ribosome biogenesis defect and general mRNA degradation.

## 4. Materials and Methods

### 4.1. Bacterial Strains and Growth Conditions

The bacterial strains and plasmids are listed in Table 2. *P. putida* strains used are derivatives of PaW85 [87], which is isogenic to the fully sequenced strain KT2440 [88]. Bacteria were grown in lysogeny broth (LB) medium. If selection was necessary, the growth medium was supplemented with kanamycin (50 μg mL^−1^). Unless noted otherwise, *E. coli* was incubated at 37 °C and *P. putida* at 30 °C. Electrotransformation was carried out as described in Reference [89].

### 4.2. Construction of Plasmids and Strains

The oligonucleotides used in PCR amplifications are listed in Table S6. For the generation of deletion strains, the pEMG-based plasmids were constructed according to a previously described protocol [90]. The upstream and downstream regions (about 600 bp) of the gene to be deleted were amplified separately and then joined into one fragment by overlap extension PCR. For the construction of the plasmid pEMG-Δ*deaD*, the 1180 bp PCR fragment was cut with KpnI and SacI. For the construction of pEMG-Δ*csiD* and pEMG-Δ*lhgO*, the 1200 and 1158 bp PCR fragments, respectively, were cut with BamHI and EcoRI and ligated into the corresponding sites of the plasmid pEMG. The intactness of the insert was checked by sequencing and the plasmids were delivered to *P. putida* wild-type and Δ*graA* strains by electroporation. After 2.5 h of growth in LB medium, the bacteria were plated onto LB agar supplemented with kanamycin. The kanamycin-resistant cointegrates were selected and electrotransformed with the plasmid pSW(I-SceI). For cointegrate resolution, the plasmid-encoded I-SceI was induced overnight with 1.5 mM 3-methylbenzoate. Kanamycin-sensitive colonies were selected and the gene deletions were verified by PCR and sequencing. The plasmid pSW(I-SceI) was eliminated from the deletion strains by growing them overnight in LB medium without antibiotics.

### 4.3. Proteomics

Bacteria for proteome analysis were grown in 50 mL LB medium with shaking at 180 rpm at both 30 °C and 25 °C. Cells were harvested in mid-log phase (OD_580_ ≈ 1.0) from three independent cultures per strain and temperature. Label-free quantification of whole cell proteomes was performed in the Proteomics Core Facility, Institute of Technology, University of Tartu, Estonia. Data analysis was performed with the Perseus software [92]. Across all samples, a total of 3012 proteins were identified. Parallel samples were grouped together and groups were compared in pairs: (i) *P. putida* wild-type (wt) at 30 °C vs. 25 °C (2561 proteins); (ii) Δ*graA* at 30 °C vs. 25 °C (2587 proteins); (iii) wt vs. Δ*graA* at 30 °C (2601 proteins); and (iv) wt vs. Δ*graA* at 25 °C (2557 proteins). To be included in the analysis, a protein needed to be detected in all three parallels of one group. Thereafter, missing values were imputed using default settings. Mean protein abundances were compared between two groups using the independent samples Student’s T-test. Benjamini-Hochberg multiple testing correction was applied with the false discovery rate set to 0.05.

To find differentially regulated transcriptional units, we analyzed the 1085 operons defined in the DOOR prediction database [57]. We searched for operons where at least 2 proteins had valid abundance values in the proteome comparison and the mean fold difference of all the measured proteins in the operon between strains was more than 2. From those, we additionally discarded two-gene operons where one had changed less than 1.5-fold.

### 4.4. Metabolomics

Bacteria for metabolomic analysis were grown identically to the proteomics analysis, except in 8 parallels per strain and temperature. Targeted metabolomics was carried out with selected hydroxyl acids, all proteogenic amino acids and acylcarnitines using a previously described method [93]. Acylcarnitines (free, acetyl-, propionyl- and butyrylcarnitine) were analyzed as the precursors of *m*/*z* 85 ion and all amino acids were analyzed by multiple reaction monitoring scan. Ionization was performed at 4500 V and 400 °C, the declustering potential was set at 40 V and collision energy was set at 38 V. For analysis of hydroxy acid and hexoses (phospho-, bisphospho- and monohexoses, lactate, succinate, citrate and oxaloacetate), 30 μL of sample was mixed with 60 μL (500 μM [^2^H_4_] succinic acid in methanol). The samples were centrifuged for 15 min at 10000 × *g* and 20 μL were analyzed by liquid chromatography-mass spectrometry QTRAP 4500 (AB Sciex, Canada). Mean metabolite abundances were compared using the independent samples Student’s T-test. Benjamini-Hochberg multiple testing correction was applied with the false discovery rate set to 0.05.

### 4.5. Temperature Tolerance Plate Assay

Cold tolerance was evaluated on LB agar plates. Serial 10-fold dilutions of LB-grown overnight cultures were spotted onto plates as 5 μL drops and incubated at different temperatures for 24 h.

### 4.6. Sucrose Gradient Profiles

Strains were grown in 75 mL liquid LB medium at 25 °C to exponential growth phase (OD_580_ ≈ 1.0). Cells were harvested by centrifugation and washed with 3 mL LLP buffer (10 mM Tris pH 8.0; 60 mM KCl; 60 mM NH_4_Cl; 12 mM MgOAc; 6 mM β-ME), pelleted again and frozen in liquid N_2_. To obtain sucrose gradient profiles, the cells were thawed on ice and prepared for lysis by 30-min incubation with 10 U/mL DNase I and 1.5 mg/mL of lysozyme. Bacteria were then lysed using 0.1 mm glass beads on a Precellys 24 homogenizer (Bertin Technologies) at 4 °C. The debris was pelleted at 16,000 *g* for 15 min at 4 °C. The OD_260_ of the supernatant was determined on a NanoDrop 2000c spectrophotometer (Thermo Scientific). 30 A260 units were loaded onto each 15%–40% (*w*/*w*) sucrose gradient in LLP buffer and centrifuged in a Beckman Coulter Sw 28 Ti rotor for ω^2^t = 2.7 ∙ 10^11^. Gradient profiles were obtained by continuous monitoring at 260 nm.

## Figures and Tables

**Table 1:** Proteins altered in the *P. putida* Δ*graA* strain at 30 °C and 25 °C.

Δ*graA* vs. WT, 30 °C				
Locus	Gene	Protein	Fold Change *	*p*-Value
PP_2645	*mgtA*	Magnesium transporter, ATP-dependent	−2.19	0.00016
PP_2487	*aldA*	Putative aldehyde dehydrogenase	−2.01	0.00028
PP_3760	*cheR3*	Putative methyltransferase CheR3	2.23	0.00024
PP_1868	*deaD*	ATP-dependent RNA helicase DeaD	2.52	0.00017
PP_1433	*rnc*	Ribonuclease 3 (RNase III)	2.55	0.00043
PP_2909	*csiD*	Protein CsiD	4.06	0.00022
**Δ*graA* vs. WT, 25 °C**				
PP_2486		NADH-dependent flavin oxidoreductase, Oye family	−3.16	0.00001
PP_0988	*gcvP1*	Glycine dehydrogenase (decarboxylating) 1	−3.16	0.00013
PP_4012	*idh*	Isocitrate dehydrogenase	−2.87	0.00002
PP_4185	*sucD*	Succinate--CoA ligase [ADP-forming] subunit alpha	−2.52	0.00005
PP_4189	*sucA*	2-oxoglutarate decarboxylase, thiamine-requiring E1 subunit	−2.12	0.00017
PP_1245		Uncharacterized protein	−2.01	0.00018
PP_1800	*rmd*	Oxidoreductase Rmd	2.01	0.00001
PP_0637 PP_4025 PP_4745	*tnpA*	Transposase	2.17	0.00003
PP_1868	*deaD*	ATP-dependent RNA helicase DeaD	3.08	2.57E-06
PP_1236		Putative Glycine cleavage system transcriptional repressor	3.68	0.00003
PP_5270	*dadA2*	D-amino acid dehydrogenase 2	5.01	0.0003
PP_1835		Uncharacterized protein	8.79	0.00014
PP_2909	*csiD*	Protein CsiD	11.08	0.00007

* More than 2-fold statistically significant changes are presented.

**Table 2:** Strains and plasmids.

Strain or Plasmid	Genotype or Characteristics	Source or Reference
*E. coli* strains		
DH5α λ*pir*	λ*pir* lysogen of DH5α	[90]
*P. putida* strains		
PaW85	Wild-type, isogenic to KT2440	[87]
Δ*graA*	*graA* is deleted from PaW85	[46]
Δ*deaD*	*deaD* (PP_1868) is deleted from PaW85	This study
Δ*graA* Δ*deaD*	*deaD* is deleted from Δ*graA* strain	This study
Δ*csiD*	*csiD* (PP_2909) is deleted from PaW85	This study
Δ*graA* Δ*csiD*	*csiD* is deleted from Δ*graA* strain	This study
Δ*lhgO*	*lhgO* (PP_2910) is deleted from PaW85	This study
Δ*graA* Δ*lhgO*	*lhgO* is deleted from Δ*graA* strain	This study
Plasmids		
pEMG	Suicide plasmid containing *lacZ*α with two flanking I-SceI sites (Km^r^)	[90]
pSW(I-SceI)	For I-SceI endonuclease expression (Ap^r^)	[91]
pEMG-Δ*deaD*	pEMG with a PCR-designed 1180 bp KpnI-SacI insert for deleting the *deaD* gene (Km^r^)	This study
pEMG-Δ*csiD*	pEMG with a PCR-designed 1200 bp EcoRI-BamHI insert for deleting the *csiD* gene (Km^r^)	This study
pEMG-Δ*lhgO*	pEMG with a PCR-designed 1158 bp EcoRI-BamHI insert for deleting the *lhgO* gene (Km^r^)	This study

## Supplementary Materials

The following are available online at http://www.mdpi.com/2072-6651/11/2/103/s1, Table S1: List of proteins identified at 30 °C and analysis of differences in protein abundance between Δ*graA* and *P. putida* wild-type. Table S2: List of proteins identified at 25 °C and analysis of differences in protein abundance between Δ*graA* and *P. putida* wild-type. Table S3: Operons with more than 2-fold mean fold change in Δ*graA* compared to *P. putida* wild-type. Table S4. List of ribosome biogenesis factors and carbon metabolism enzymes visualized in Figure 3. Table S5. Comparison of metabolite levels in Δ*graA* and wild-type *P. putida*. Table S6. Oligonucleotide sequences used in this study. Figure S1. Growth curves of *Pseudomonas putida* wild-type and Δ*graA* cultures used in proteome analysis. Figure S2. Volcano plots representing the comparisons of the *P. putida* Δ*graA* strain to the wild-type at 30 °C and 25 °C with all ribosomal proteins highlighted.
