# Supplementary material for: Pseudomonas putida Responds to the Toxin GraT by Inducing Ribosome Biogenesis Factors and Repressing TCA Cycle Enzymes
Source: Toxins (Basel). 2019 Feb 9;11(2):103. doi: 10.3390/toxins11020103 (PMC6410093; doi:10.3390/toxins11020103)
Supplement: Supplementary file 1 [file toxins-11-00103-s001.zip › toxins-422469-supple-final/toxins-422469-supple-final.docx]

Supplementary Materials: *Pseudomonas putida* Responds to the Toxin GraT by Inducing Ribosome Biogenesis Factors and Repressing TCA Cycle Enzymes

Andres Ainelo, Rando Porosk, Kalle Kilk, Sirli Rosendahl, Jaanus Remme and
Rita Hõrak


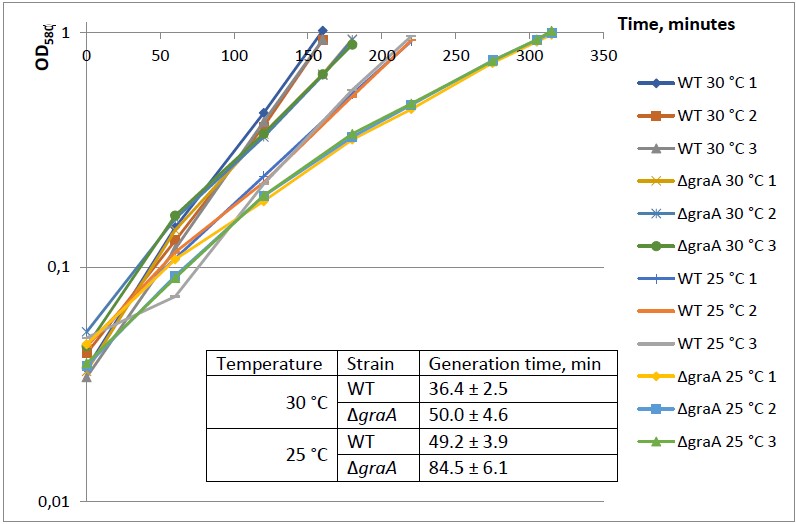


**Figure S1.** Growth curves of Pseudomonas putida wild-type and ΔgraA cultures used in proteome analysis. Bacteria were grown in 50 mL LB medium at indicated temperatures. Bacteria for generation time determination were grown independently at analogous conditions with OD580 measurements at 30-minute intervals. Generation times were calculated from the slope of the exponential growth according to the formula G=t/3.3log(b/B), where G marks the generation time, t – the time interval in minutes, B and b – OD580 at the beginning and the end of the time interval, respectively. Averages of three independent cultures with 95% confidence intervals are presented.


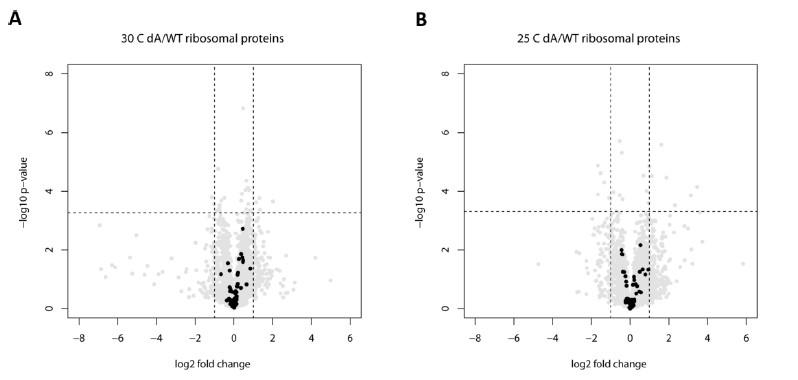


**Figure 2.** Volcano plots representing the comparisons of the P. putida ΔgraA strain to the wild-type at 30 °C (**A**) and 25 °C (**B**) with all ribosomal proteins highlighted as black dots. Horizontal dashed lines indicate the statistical significance thresholds after Benjamini-Hochberg multiple testing correction (FDR = 0.05). Vertical dashed lines indicate twofold difference between the compared proteomes..
